# Recombinant AfusinC, an anionic fungal CSαβ defensin from *Aspergillus fumigatus*, exhibits antimicrobial activity against gram-positive bacteria

**DOI:** 10.1371/journal.pone.0205509

**Published:** 2018-10-11

**Authors:** Gabriela Contreras, Markus Santhosh Braun, Holger Schäfer, Michael Wink

**Affiliations:** Institute of Pharmacy and Molecular Biotechnology, Heidelberg University, Heidelberg, Germany; University of Manchester, UNITED KINGDOM

## Abstract

Antimicrobial peptides (AMPs) are short and generally positively charged peptides found in a wide variety of organisms. CSαβ defensins are a group of AMPs. These defensins are composed of an α-helix and a β-sheet linked by three or four disulphide bridges. In this study, we describe the antimicrobial activity of an anionic CSαβ fungal defensin from *Aspergillus fumigatus*, AfusinC. AfusinC was recombinantly produced as a fusion protein in *Escherichia coli*. The tag was removed by proteolytic cleavage, and AfusinC was purified by size exclusion chromatography. About 0.8 mg of recombinant AfusinC was obtained from 1 L of culture. Recombinant AfusinC was active against mainly gram-positive bacteria including human pathogens and a multiresistant-strain of *A*. *aureus*. Additionally, AfusinC showed bactericidal effect against *Micrococcus luteus*.

## Introduction

Overuse of antibiotics has caused the development of new multidrug-resistant microorganisms; as a result, it is estimated that each year 25,000 patients die due to the most common multidrug-resistant bacteria in Europe (i.e. *Staphylococcus aureus*, *Enterobacteriaceae*, *Pseudomonas aeruginosa*) [1]. Hence, it is important to search new antimicrobial agents to overcome this problem. A group of antimicrobial compounds, naturally synthesised by organisms, are antimicrobial peptides (AMPs). AMPs are produced in both prokaryotes and eukaryotes. AMPs are part of the innate immune systems in multicellular organisms [2], for this reason, they are also called host defence peptides. AMPs are short peptides (12 to 50 amino acids), commonly cationic, and amphipathic. These characteristics enhance selective attachment and insertion of AMPs onto the negatively charged microbial cytoplasmic membranes [3]. This insertion can lead to leaky biomembranes or membrane disruption and consequently to death of microbial cells. AMPs are considered a promising alternative to common antibiotics because they exhibit a broad-spectrum activity against bacteria, fungi, parasites, and viruses. Additionally, the appearance of AMPs-resistant strains is less likely [4].

The largest group of AMPs are defensins [5]. These peptides are cysteine-rich, amphipathic, and positively charged. Defensins are found in a wide range of organisms such as vertebrates, invertebrates, plants, and fungi. In arthropods, plants, and fungi, defensins are composed of an α-helix linked to an antiparallel two-stranded β-sheet by disulphide bridges; this is called a cysteine-stabilised α-helix/β-sheet (CSαβ motif). CSαβ is a common motif in peptides with antimicrobial activity [6].

Fungi are attractive sources of antimicrobial compounds. The first fungal CSαβ defensin identified was plectasin which is produced by the saprophytic ascomycete *Pseudoplectania nigrella* [7]. To date, eurocin from *Eurotium amstelodami* [8] and micasin from *Microsporum canis* [9] have been studied regarding their structures, functions, and therapeutic potential. Regarding the mechanism of action, it was found that plectasin, and other CSαβ defensins, inhibit peptidoglycan synthesis of predominantly gram-positive bacteria by binding to lipid II [8, 10].

Based on sequence similarity and the presence of the CSαβ motif, eight families of fungal defensins have been predicted from published fungal genomes [11, 12]. Among them, they described AfusinC, a putative anionic fungal defensin from the fungus *Aspergillus fumigatus*. This fungus is an opportunistic human pathogen and is the leading cause of fungal infections in immunosuppressed patients.

In the present study, we report on the antimicrobial activity of AfusinC produced as a recombinant peptide in *Escherichia coli*. AfusinC was expressed as a fusion protein with thioredoxin (Trx). Fusion partner was removed by proteolytic cleavage, and subsequently, AfusinC was purified by size exclusion chromatography. AfusinC was characterised in relation to its antimicrobial activity and haemolytic activity.

## Materials and methods

### Bacterial strains and growth conditions

*E*. *coli* BL21(DE3) (New England Biolabs, USA) was used as an expression host for protein expression. *E*. *coli* BL21(DE3) was grown with constant agitation at 37 °C in Luria-Bertani (LB) medium.

Antimicrobial activity was studied against gram-positive bacteria (*Bacillus subtilis* DSM 10, *Bacillus megaterium* DSM 32, *Micrococcus luteus* DSM 20030, *Staphylococcus auricularis* DSM 20609, *Enterococcus faecalis* ATCC 29212, *Staphylococcus aureus* ATCC 25923, methicillin-resistant *S*. *aureus* (MRSA) NCTC 10442, and *Streptococcus pyogenes* ATCC 1234), and gram negative-bacteria (*E*. *coli* K12 DSM 498, *Pseudomonas fluorescens* DSM 50090, *Acinetobacter bohemicus* DSM 100419, and *Yersinia mollaretii* DSM 18520). Bacteria were grown in Cation-adjusted Mueller-Hinton Broth (MHB) (Sigma, USA) or Brain Heart Infusion Broth (BHI) (Sigma, USA) at 30 or 37 °C.

Bacterial strains were obtained from the German Collection of Microorganisms and Cell Cultures (DSMZ). *Enterococcus faecalis* ATCC 29212, *Staphylococcus aureus* ATCC 25923, MRSA NCTC 10442, and *Streptococcus pyogenes* ATCC 12344 were supplied by the Department of Infectious Diseases, Medical Microbiology and Hygiene, Heidelberg University, Heidelberg, Germany.

### Construction of pET-32c_afuC plasmid

Defensin domain protein of *Aspergillus fumigatus* Af293 (GenBank accession: EAL86953.1) was analysed by SignalP 4.1 Server [13] and ProP 1.0 Server (http://www.cbs.dtu.dk/services/ProP/) [14] to predict signal peptide cleavage and pro-peptide cleavage sites, respectively. The DNA sequence coding for AfusinC (*afuC* gene) was synthesised by Eurofins Genomics (Germany). This DNA sequence was optimised for codon usage in *E*. *coli*. A TEV cleavage site was added in 5' end of the *afuC* gene and two stop codons (TAA) in the 3' end. This DNA sequence was cloned into the pET-32c(+) vector (Merck, Germany) using *XhoI* and *EcoRI* restriction sites. The resulting recombinant DNA contained sequentially in-frame a *trxA* gene, an internal hexahistidine (His6) tag, a TEV protease recognition site, and an *afuC* gene. The obtained plasmid was called pET-32c_afuC which confers ampicillin resistance.

### Expression and purification of fusion protein, Trx-AfusinC

The pET-32c_afuC plasmid was transformed into *E*. *coli* BL21(DE3). To express Trx-AfusinC, a single colony was inoculated in LB medium supplemented with 100 μg/mL ampicillin; it was grown at 250 rpm, 30 °C, overnight. This culture was used to inoculate 500 ml LB medium supplemented with 100 μg/mL ampicillin. The bacterial culture was grown at 250 rpm at 37 °C until it reached an optical density at 600 nm of 0.7. The protein expression was induced with 0.5 mM isopropyl-β-D-thiogalactopyranoside (IPTG) (AppliChem GmbH, Germany) for 3 h at 37 °C. The bacterial cells were collected by centrifugation and suspended in lysis buffer (50 mM NaH_2_PO_4_, 300 mM NaCl, 1 mM DTT, pH 8) (10 ml/1 g wet weight of cell). The cells were lysed adding 1 mg/ml lysozyme (AppliChem GmbH, Germany) for 30 min at 4 °C, followed by sonication at 50% for 5 min (Omni Ruptor 4000 Ultrasonic Homogenizer, Omni International, USA). The bacterial lysate was centrifuged at 15,000 g for 20 min at 4 °C, and the pellet containing the fusion protein was collected. Inclusion bodies were washed with lysis buffer containing 1% Triton X-100 and then with lysis buffer. The inclusion bodies were solubilised with denaturing solubilisation buffer (50 mM NaH_2_PO_4_, 300 mM NaCl, 8 M urea, 1 mM DTT) for 1 h.

The fusion protein was purified under denaturing conditions using an ÄKTA Start chromatography system (GE Healthcare Life Sciences, USA) by immobilised metal affinity chromatography (IMAC). The solubilised inclusion bodies were applied onto a Ni-IDA column (Bio-Scale Mini Profinity IMAC Cartridges, Bio-Rad, USA). The column was washed with denaturing solubilisation buffer containing 5 mM imidazole, and the fusion protein was eluted in 250 mM imidazole. The fractions containing Trx-AfusinC were pooled and refolded *in vitro* by dialysis in two-steps: 50 mM Tris-HCl, 0.5 mM EDTA, 3 mM reduced glutathione (GSH), 0.3 mM oxidized glutathione (GSSG), 3 M urea, pH 8, and then, 50 mM Tris-HCl, 0.5 mM EDTA, pH 8. Each dialysis step lasted for 24 h and was performed in a dialysis tubing (VISKING, Serva, Germany) at 4 °C.

The protein concentration was determined according to Bradford [15] using bovine serum albumin (BSA) as standard. The fusion protein was checked by 12% glycine sodium dodecyl sulphate polyacrylamide gel electrophoresis (SDS-PAGE), and it was stained with Coomassie blue R-250.

### Proteolytic cleavage and purification of AfusinC

The fusion protein was cleaved by cysteine protease from tobacco etch virus (TEV) (Protean, Czech Republic) (1:100 w/w) in TEV cleavage buffer (50 mM Tris, 0.5 mM EDTA, 1 mM DTT, pH 8). The reaction was incubated overnight at 4 °C. The cleavage was analysed by 16% Tricine-SDS-PAGE gel [16], and it was stained by silver blue staining [17]. The percentage cleavage was calculated based on SDS-PAGE gel using ImageJ software [18].

AfusinC was purified by size exclusion chromatography with a HiPrep Sephacryl S-100 16/60 column (GE Healthcare Life Sciences, USA) in 50 mM Tris-HCl, 0.5 mM EDTA, pH 8, at a flow rate of 0.5 ml/min by an ÄKTA Start chromatography system (GE Healthcare Life Sciences, USA). For bioactivity studies, the buffer was exchanged against phosphate-buffered saline (PBS) by a PD-10 desalting column (GE Healthcare Life Sciences, USA).

The molecular weight of the recombinant AfusinC was analysed by liquid chromatography–mass spectrometry (LC-MS) at the Core Facility for Mass Spectrometry and Proteomics, ZMBH (Center for Molecular Biology of Heidelberg University), Heidelberg, Germany.

Purified AfusinC was quantified by spectrophotometry at 280 nm using its extinction coefficient 7,490 M^-1^ cm^-1^. The extinction coefficient was determined from its amino acid sequence using the program ProtParam [19].

### Circular dichroism

Far-UV circular dichroism (CD) spectrum of AfusinC was recorded on Jasco J-715 CD spectrometer (Japan) in a 0.1 cm cuvette at room temperature. The spectrum of 50 μM AfusinC was obtained in 10 mM potassium phosphate buffer (pH 7.6). The spectra were recorded and corrected for the blank. CD data are expressed as millidegrees (mdeg).

### Antimicrobial activity

The minimum inhibitory concentration (MIC) was determined by the broth microdilution method according to Wiegand et al. [20]. Briefly, 4–5 fresh bacterial colonies were suspended in 0.9% NaCl to a turbidity of 0.5 McFarland. The suspension was diluted 1:100 in MHB or BHI. AfusinC and antibiotics were serially diluted to obtain 64–0.125 μg/ml of the final concentration. Ampicillin and vancomycin were used as a positive control, and PBS as a negative control. The 96-well plates were incubated at 30 or 37 °C overnight, according to the optimum growth conditions of the strain. The MIC was defined as the lowest concentration without visible growth. Following MIC determination, minimum bactericidal concentration (MBC) was evaluated by plating 10 μl samples from wells with no visible growth onto LB agar plates. After overnight incubation, MBC was defined as the lowest concentration that produced at least 99.9% reduction of the original inoculum (S1 Fig). Three independent experiments were performed for MIC and MBC determination.

### Time-kill curve

Time-kill curve was established based on Clinical and Laboratory Standards Institute (CLSI) [21]. Briefly, the bacterial cultures were incubated in MHB at 37 °C until 0.5 McFarland and then diluted 10 times in MHB. The cultures were incubated in 1X, 2X, 4X MIC of AfusinC or without peptide as growth control in 200 μl MHB at 37 °C. Aliquots (30 μl) were withdrawn at 0, 3, 6, 12, 24, and 48 h, then serially diluted. 10 μL of each dilution (in duplicate) were spotted onto LB agar plates. The number of colony-forming units (CFU) was counted after 2 days of incubation at 37 °C. The results represent the average of three independent experiments. Bactericidal activity was considered a decrease of 3log_10_ CFU/mL. Statistical analyses were performed using GraphPad Prism version 5.

### Haemolysis assay

Defibrinated sheep blood (Thermo Fisher Scientific, USA) was washed 4 times with PBS at 900 g for 10 min. Recombinant AfusinC was diluted to obtain 200–0.1 μg/ml final concentrations in 2% erythrocytes. 0.5% Triton X-100 and PBS were used as positive and negative controls, leading to 100% and 0% of lysed cells, respectively. The solution was incubated at 37 °C for 60 min. The plates were centrifuged at 2,000 g for 5 min, and the supernatant was collected. The absorbance of the supernatant was measured at 554 nm using a TECAN spectrophotometer (Infinite 200 PRO NanoQuant, Switzerland). The results represent the average of three independent experiments.

## Results

### Production and purification of Trx-AfusinC

**Fig 1:**
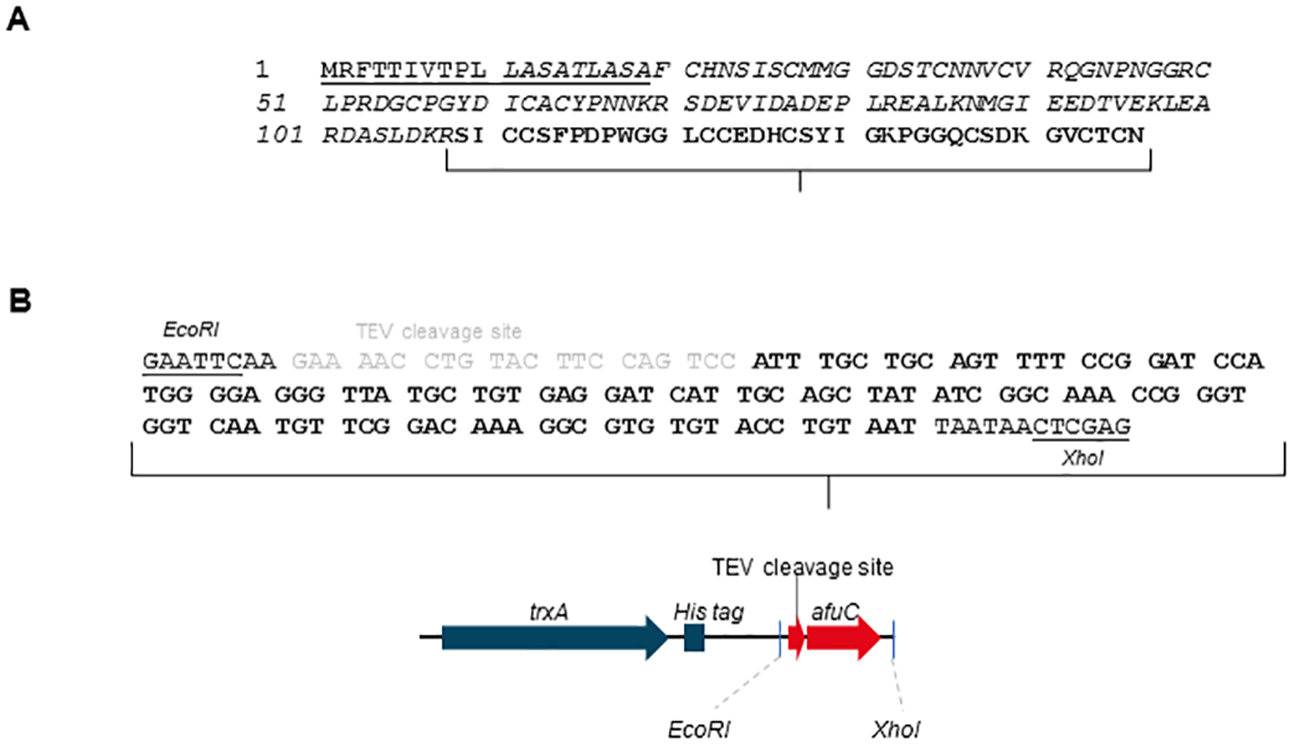
Cloning of *afuC* gene into pET-32c (+). (A) Defensin domain protein produced by *Aspergillus fumigatus* (GenBank: EAL86953.1): Predicted signal peptide region is underlined (1–19), pro-peptide in italic (20–108), and AfusinC in bold (predicted mature peptide, 109–146). (B) Nucleotide sequence coding AfusinC was synthetically synthesised. Restriction sites, *EcoRI* and *XhoI*, were included for cloning into pET-32c(+) vector. Additionally, a TEV cleavage site was incorporated for the further release of AfusinC. The nucleotide sequence encoding AfusinC and a TEV cleavage site are bold and grey, respectively. DNA sequence was optimised for codon usage in *E*. *coli*. The synthesised sequence was cloned into pET-32c(+) vector using *EcoRI* and *XhoI* sites, downstream from the *trxA* gene and His tag.

The amino acid sequence of the defensin domain protein produced in *A*. *fumigatus* (XP_748991) was analysed to identify the putative signal sequence and pro-peptide. According to the cleavage sites, the mature peptide was identified as indicated in Fig 1A. The mature peptide predicted was named AfusinC by Zhu [11] who previously identified it as a putative CSαβ defensin.

In order to express a recombinant AfusinC in *E*. *coli*, the pET-32c_afuC plasmid was built (Fig 1B). To avoid the lethality of AfusinC to *E*. *coli*, *afuC* gene was fused to the carboxy-terminus of thioredoxin (Trx) as a fusion partner whose gene is contained in pET-32c(+) vector. Additionally, between *trxA* gene and *afuC* gene, a sequence coding a cleavable TEV protease site was added for the later release of the AfusinC.

**Fig 2:**
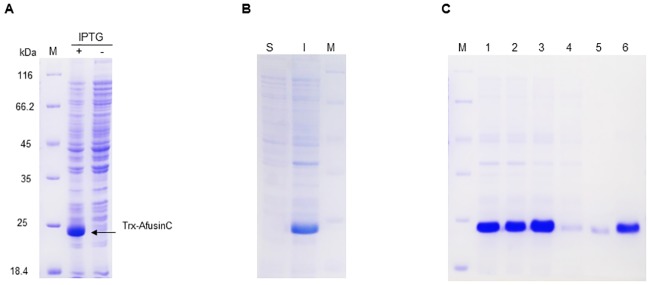
Purification of Trx-AfusinC. (A) SDS-PAGE analysis of the recombinant fusion protein (Trx-AfusinC) in *E*. *coli*. Expression *E*. *coli* BL21(DE3) harbouring pET-32c_afuC was induced with IPTG (+). The crude extract of induced culture is compared with non-induced culture (-). (B) The crude extract was centrifuged, and soluble (S) and insoluble (I) fractions were analysed. (C) Insoluble fraction was collected (1). Then, Inclusion bodies were washed with 1% Triton X-100 (2) and solubilised with 8 M urea (3). Solubilised Trx-AfusinC was purified by IMAC (Ni-IDA). Flow-through (4), wash (5), and elution (6) fractions are shown. Protein samples were separated on 12% Glycine SDS-PAGE gel which was stained with Coomassie blue R-250. The relative molecular weights expressed in kDalton (kDa) of the protein marker (M) are shown.

The pET-32c_afuC plasmid was expected to encode a fusion protein, Trx-AfusinC, with a molecular weight of 23 kDa, composed by Trx (109 residues), His6 (6 residues), TEV recognition (6 residues) and AfusinC (38 residues). The pET-32c_afuC was transformed into *E*. *coli* BL21(DE3). Under the induction by IPTG, a protein with the expected molecular weight was produced (23 kDa, Fig 2A) which was accumulated as insoluble inclusion bodies, reaching approximately 80% of the total bacterial protein (Fig 2B). The inclusion bodies were solubilised in the presence of 8 M urea, and the fusion protein was purified under denaturing conditions by IMAC (Fig 2C). The fusion protein was refolded *in vitro* by dialysis in two steps using GSSG and GSH (molar ratio 1:10) as redox reagents for disulphide bridges formation in a Tris buffer, pH 8.

### Production and purification of AfusinC

**Fig 3:**
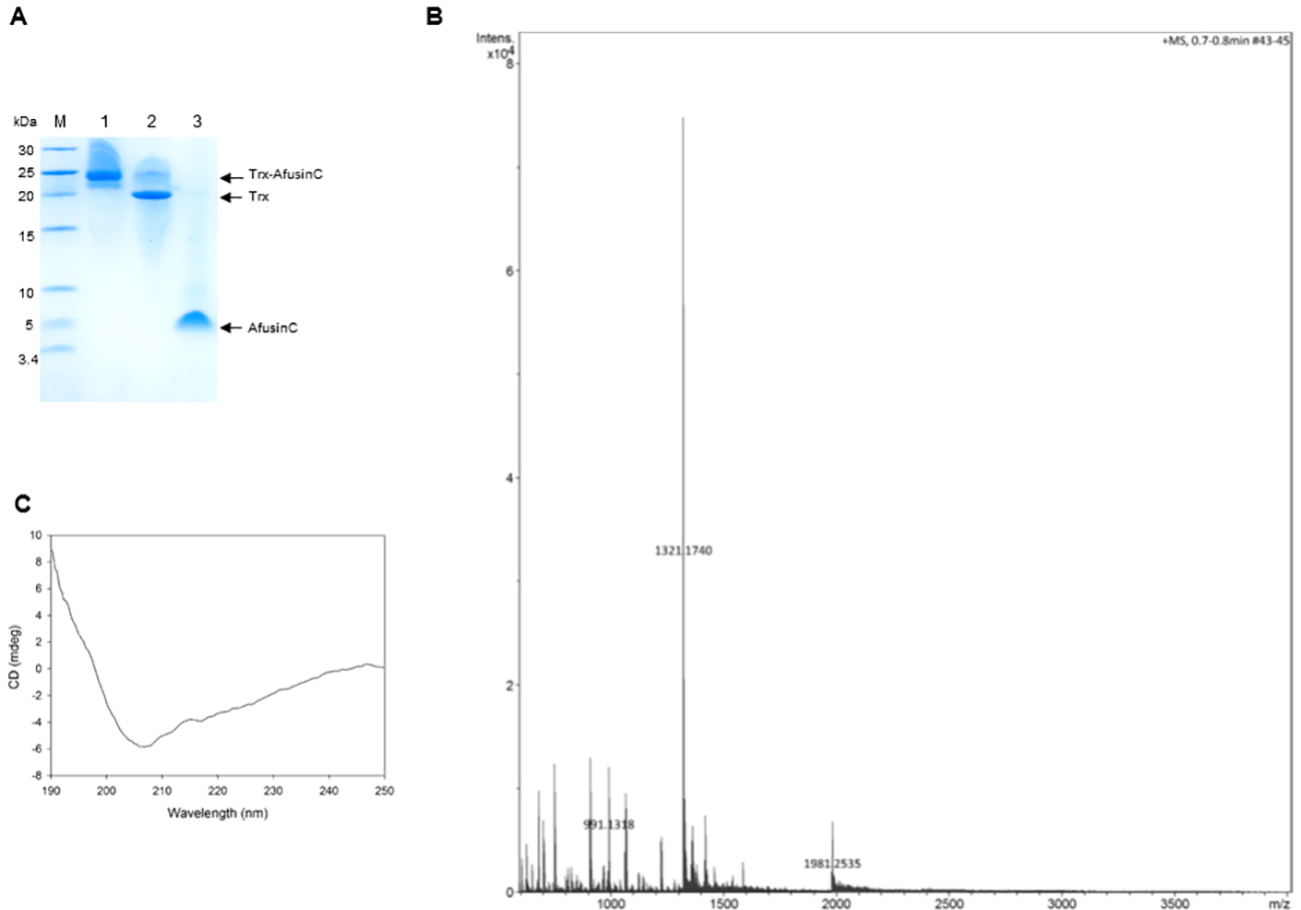
Purification, MS and CD analysis of AfusinC. (A) Tricine- SDS-PAGE analysis of AfusinC purification. Lanes 1, Refolded Trx-AfusinC; 2, Trx-AfusinC cleaved by TEV protease; 3, AfusinC purified by size exclusion chromatography. Samples were separated on 16% Tricine-SDS-PAGE. Gel was stained with silver blue staining. (B) Mass spectrum of the recombinant AfusinC. The ions at *m/z* 991.132, 1321.174, and 1981.254 are corresponding to the expected monoisotopic products of fully oxidised AfusinC (3960.490 Da). (C) Far-UV CD spectrum of AfusinC. 50 μM AfusinC in 10 mM potassium phosphate buffer (pH 7.6) at room temperature. The spectrum shown is an average of three scans.

The recombinant AfusinC was obtained by the cleavage of the refolded fusion protein with TEV protease (Fig 3A). The efficiency of the cleavage was 88 ± 3%. The recombinant AfusinC was subsequently purified by size exclusion chromatography. Tricine-SDS-PAGE of purified AfusinC revealed a band with the expected molecular weight (~4 kDa).

The identity of AfusinC was confirmed by LC-MS (Fig 3B). The mass spectrum shows ions at mass-to-charge ratio (*m/z)* 991.132, 1321.174, and 1981.254. These values correspond to the monoisotopic expected products, (M + 2H)^2+^, (M + 3H)^3+^, (M + 4H)^4+^, respectively, for fully oxidised AfusinC (monoisotopic mass 3960.490 Da).

The purified AfusinC was analysed by CD spectroscopy (Fig 3C). The far-UV CD spectrum of AfusinC displayed a minimum at 208 nm corresponding to α-helix and a signal at 218 nm for β-sheet, indicating a well-folded CSαβ defensin. The final yield of AfusinC was 0.8 mg/L of bacterial culture. The purification steps are summarised in Table 1.

**Table 1:** Purification steps of AfusinC from a 1L culture of *E*. *coli* BL21(DE3) harbouring the plasmid pET-32c-afuC.

Purification step	Total protein (mg)^a^	Trx-AfusinC (mg)	AfusinC (mg)	Yield (%)
Solubilized inclusion bodies	66	53 (81)	9^b^ (13)	100
Ni-IDA affinity chromatography	24	23 (96)	4^b^ (17)	44
Cleavage of Trx-AfusinC by TEV protease and size exclusion chromatography	0.9	ND	0.8 (88)	8.8

Numbers in parentheses correspond to the percentage in relation to the total protein content.

^a^Total protein quantified by Bradford using BSA as standard.

^b^The amount of AfusinC was estimated in relation to the mass-ratio of the fusion protein.

ND: Not determined.

### Antimicrobial activity

Antimicrobial activity was carried out by broth microdilution method against gram-positive and gram-negative bacteria. Unexpectedly, the uncleaved fusion protein showed antimicrobial activity but only against *M*. *luteus* (MIC = 32 μg/ml). Regarding the isolated AfusinC, the MIC and MBC values are shown in Table 2. AfusinC was active against most of the gram-positive bacteria tested including human pathogens and a multi-resistant strain (MRSA) with a MIC ranging from 4 to 64 μg/ml. Most potent activities were detected against *B*. *megaterium* and *M*. *luteus* with a MIC of 4 μg/ml; while in gram-negative bacteria, it showed antimicrobial activity only in *A*. *bohemicus* and *Y*. *mollaretti*, with MIC values of 32–64 μg/ml.

**Table 2:** Antimicrobial activity of recombinant AfusinC determined by broth microdilution.

	AfusinC		Ampicillin	Vancomycin
Strain	MIC (μg/ml)	MBC (μg/ml)	MIC (μg/ml)	MIC (μg/ml)
**Gram-positive**				
*Bacillus subtilis* DSM 10	16	64	0.25	0.25
*Bacillus megaterium* DSM 32	4	16	1	0.5
*Micrococcus luteus* DSM 20030	4	4	0.25	0.25
*Enterococcus faecalis* ATCC 29212	>64	>64	4	16
*Staphylococcus auricularis* DSM 20609	16	32	0.25	0.25
*Staphylococcus aureus* ATCC 25923	8	16	1	2
MRSA NCTC 10442	16	32	16	2
*Streptococcus pyogenes* ATCC 12344	64	64	≤0.25	2
**Gram-negative**				
*Escherichia coli* K12 DSM 498	>64	>64	16	>64
*Pseudomonas fluorescens* DSM 50090	>64	>64	>64	>64
*Acinetobacter bohemicus* DSM 100419	32	64	4	4
*Yersinia mollaretii* DSM 18520	64	>64	>64	>64

MBC values were evaluated in the bacteria studied, whose values are 1- to 4-fold of the corresponding MIC values, suggesting a bactericidal effect of AfusinC in gram-positive bacteria.

**Fig 4:**
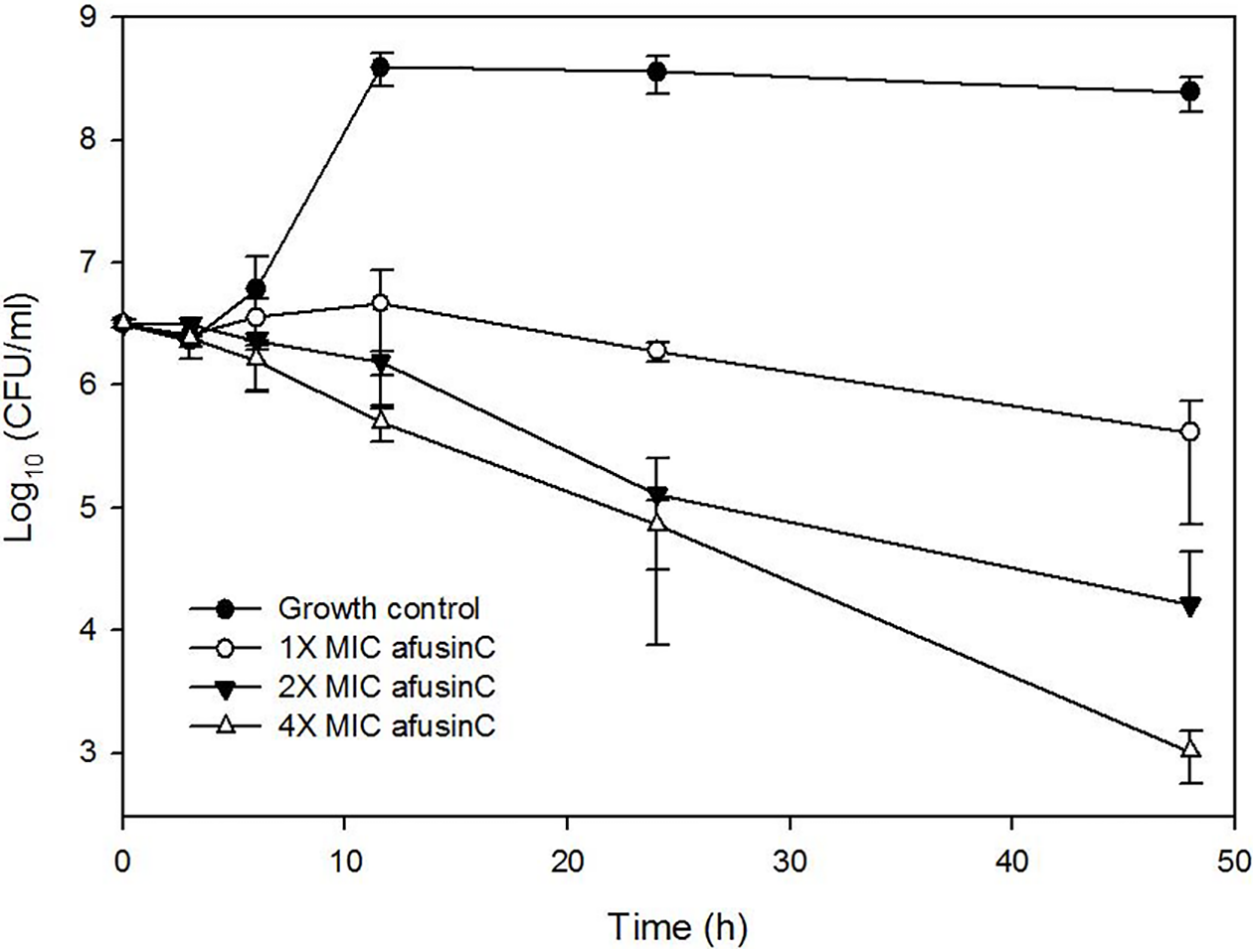
Time-kill curve of *M*. *luteus* DSM 20030 by AfusinC. *M*. *luteus* at exponential-phase was grown in MHB at 37 °C with 3 different concentrations of AfusinC: 1X MIC (4 μg/ml), 2X MIC (8 μg/ml), and 4X MIC (16 μg/ml). Bacterial inoculum without AfusinC was included as growth control. The data are shown as the means ± standard deviations (SD) of three independent experiments.

A time-kill study was performed against *M*. *luteus* (gram-positive) at 1X, 2X to 4X MIC (Fig 4). Statistical difference of bacterial concentration was observed after 12 h of incubation (p<0.001, 2-way ANOVA with Bonferroni post-test) in the presence of AfusinC (1X, 2X, and 4X MIC) in comparison to the control without the fungal CSαβ defensin. At 1X MIC after 24 h of incubation, a significant decrease relative to the original culture was observed (p<0.01, Student’s t-test). Regarding 4X MIC at 48 h, the culture showed a reduction in 3log_10_ of bacterial concentration when compared to the original culture supporting the bactericidal effect of AfusinC.

### Haemolytic activity

Several AMPs have been reported to exhibit high haemolytic activity. Haemolytic activity of recombinant AfusinC was tested on sheep erythrocytes. No haemolysis was observed at concentrations bellow 50 μg/ml. The use of 100 μg/ml and 200 μg/ml AfusinC caused 3.0 ± 0.8 and 55 ± 8% of haemolysis, respectively.

## Discussion

AfusinC was recombinantly expressed in *E*. *coli* which is the most widely used host for heterologous protein engineering. To prevent the toxicity in the host, a Trx tag was added. Trx promotes target protein solubility and catalyses the formation of disulphide bridges in the *E*. *coli* cytoplasm [22]. Trx has been successfully used as a fusion partner to express soluble recombinant cysteine-rich AMPs in *E*. *coli* BL21(DE3) [23, 24]. However, tags do not function equally with all partner proteins [25]. In this study, Trx-Afusin was predominantly expressed as inclusion bodies in *E*. *coli* BL21(DE3), even at low incubation temperature and IPTG concentration (S2 Fig). Additionally, pET-32c_afuC was transformed into Origami2(DE3)pLysS *E*. *coli*, a permissive strain for the formation of disulphide bonds in the cytoplasm due to mutations in both the thioredoxin reductase and glutathione reductase genes [26]. However, the production of Trx-Afusin was only 5 mg/L of the bacterial culture (S3 Fig). Nonetheless, inclusion bodies achieve high yield and purity of recombinant proteins [27], and in this study, the refolded Trx-AfusinC reached 54 mg/L of the bacterial culture. Furthermore, misfolded AMPs could mask their toxic activity and would not be susceptible to host proteases. Therefore, AfusinC may be feasible to produce without a fusion partner if it formed inclusion bodies, and it would not be lethal to the *E*. *coli* host.

CSαβ defensins are cysteine-rich peptides, and the correct folding is a general problem in cysteine-rich peptides when they are expressed in bacteria [28]. The final yield of AfusinC was ~ 0.8 mg/L of culture; similar yield obtained previously for purification of a CSαβ defensin from inclusion bodies [29]. This recombinant approach yielded enough material for antimicrobial assays. However, for large-scale production, the peptide output could be improved using another heterologous host, such as *Pichia pastoris*. *P*. *pastoris* has been successfully used to produce CSαβ defensins [30].

The fusion protein showed antimicrobial activity against *M*. *luteus* which was 8 times less compared to the purified peptide, indicating that Trx decreased the activity but did not inhibit it completely. Regarding the purified AfusinC, it was active mainly in gram-positive bacteria in accordance with other CSαβ defensins. Furthermore, a time-kill curve of AfusinC was performed against *M*. *luteus* indicating a bactericidal effect as reported in other fungal CSαβ defensins [31]. It has been reported that the target of some CSαβ defensins is lipid II, a peptidoglycan precursor [32]. The target of AfusinC might be lipid II as well; however, this mechanism of action needs further investigation.

The haemolytic activity is one of the drawbacks for the clinical applications of AMPs [4]. For this reason, the haemolytic activity was studied AfusinC. The haemolysis on sheep erythrocytes was only 3% at 100 μg/ml. However, to study the potential clinical application of AfusinC, a cytotoxicity test has to be performed.

We described the characterisation of a novel anionic fungal defensin. To date, most CSαβ defensins characterised are cationic peptides [33]. However, more than 60% of the putative fungal CSαβ defensins are anionic [11]. Probably not all of them are involved in the defence response because, despite their CSαβ motif, some of them have been found to lack antibacterial activity [34]. Moreover, CSαβ defensins exhibit a variety of biological functions alternative to antimicrobial activity [6].

There are some reports about anionic CSαβ defensins in invertebrates [35–36], and this is the first report of antimicrobial activity of an anionic CSαβ fungal defensin. Therefore, the positive net charge would be not required for the antimicrobial activity in CSαβ defensins. It has been suggested that a positive amino acid of the loop 3 of CSαβ defensins plays a role in the antimicrobial activity [37], and this amino acid is positively charged in AfusinC (Lys-32) based on the 3D structure [11].

In summary, AfusinC was produced in *E*. *col*i as a fusion protein. The fusion protein was successfully refolding *in vitro* resulting in an active peptide after the cleavage of the fusion partner. Although AfusinC is anionic CSαβ, it showed antibacterial activity mainly against gram-positive bacteria. Therefore, the net charge would not be essential for antimicrobial activity in CSαβ defensins.

## Supporting information

S1 Fig MIC and MBC interpretation. MIC was recorded as the lowest concentration without visible growth. 10 μl from wells with no visible growth were transferred to LB agar plates. After overnight incubation, MBC was recorded as the lowest concentration that produced ≥ 99.9% reduction of the initial bacterial inoculum. -: sterility control, +: growth control.
(TIF)

S2 Fig Optimization of Trx-AfusinC expression in *E*. *coli* BL21(DE3) harbouring pET-32c_afuC plasmid. (A) Trx-AfusinC expression was induced for 3 h at 37 °C with 0.5, 0.1, or 0.05 mM IPTG. (B) Trx-AfusinC expression was induced with 0.5 mM IPTG for 3 h at 37, 30, 20, and 4 °C. The crude extract was centrifuged, and insoluble (I) and soluble (S) fractions were analysed. Protein samples were separated by 12% Glycine SDS-PAGE and visualised by Coomassie blue R-250.
(TIF)

S3 Fig Trx-AfusinC expression in *E*. *coli* Origami2(DE3)pLysS and BL21(DE3) harbouring pET-32c_afuC plasmid. Trx-AfusinC expression was induced with 0.5 mM IPTG for 3 h at 37 °C *E*. *coli* Origami2(DE3)pLysS and BL21(DE3). The crude extract was centrifuged, and insoluble (I) and soluble (S) fractions were analysed. Protein samples resolved by 12% Glycine SDS-PAGE. The gel was stained with Coomassie blue R-250.
(TIF)
