# Supplementary figures and images for: Recombinant AfusinC, an anionic fungal CSαβ defensin from Aspergillus fumigatus, exhibits antimicrobial activity against gram-positive bacteria
Source: PLoS One. 2018 Oct 11;13(10):e0205509. doi: 10.1371/journal.pone.0205509 (PMC6181372; doi:10.1371/journal.pone.0205509)

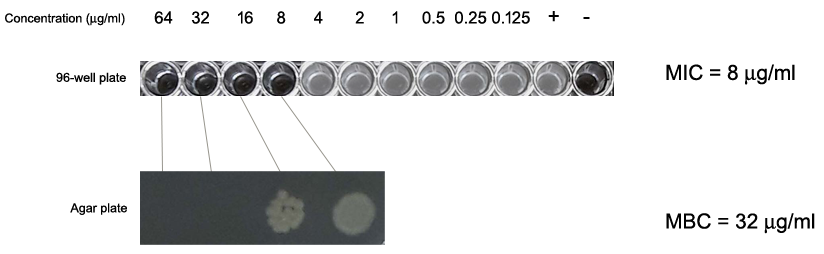

Supplement: S1 Fig — MIC was recorded as the lowest concentration without visible growth. 10 μl from wells with no visible growth were transferred to LB agar plates. After overnight incubation, MBC was recorded as the lowest concentration that produced ≥ 99.9% reduction of the initial bacterial inoculum. -: sterility control, +: growth control. (TIF) [file pone.0205509.s001.tif]

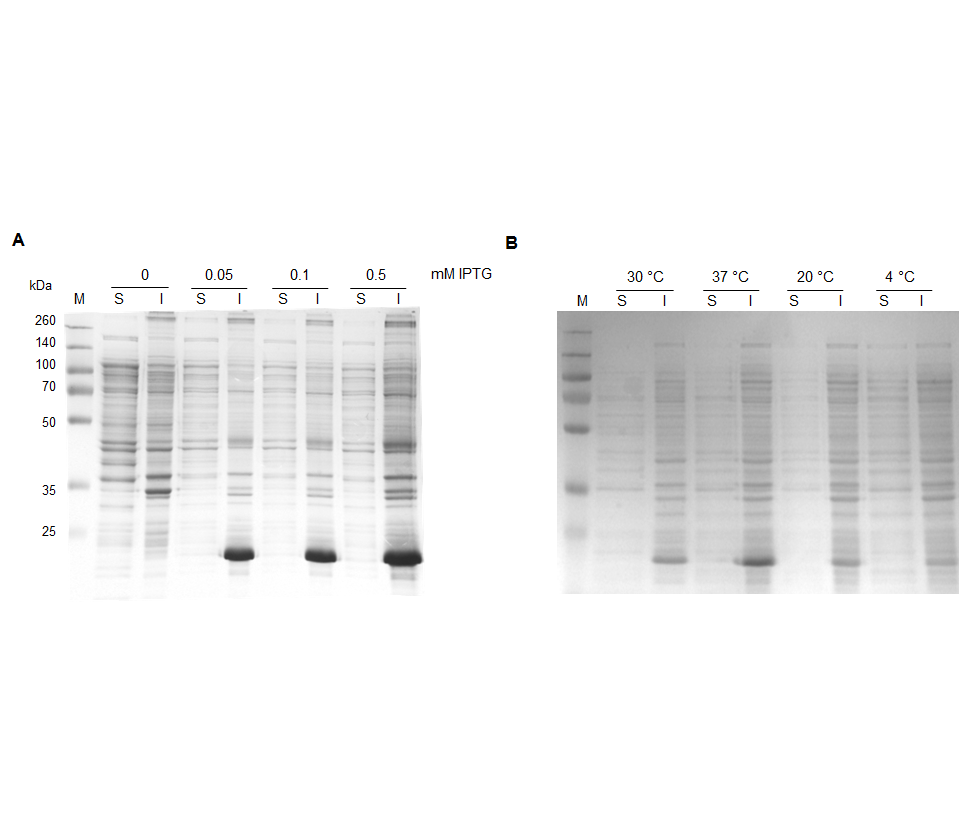

Supplement: S2 Fig — (A) Trx-AfusinC expression was induced for 3 h at 37 °C with 0.5, 0.1, or 0.05 mM IPTG. (B) Trx-AfusinC expression was induced with 0.5 mM IPTG for 3 h at 37, 30, 20, and 4 °C. The crude extract was centrifuged, and insoluble (I) and soluble (S) fractions were analysed. Protein samples were separated by 12% Glycine SDS-PAGE and visualised by Coomassie blue R-250. (TIF) [file pone.0205509.s002.tif]

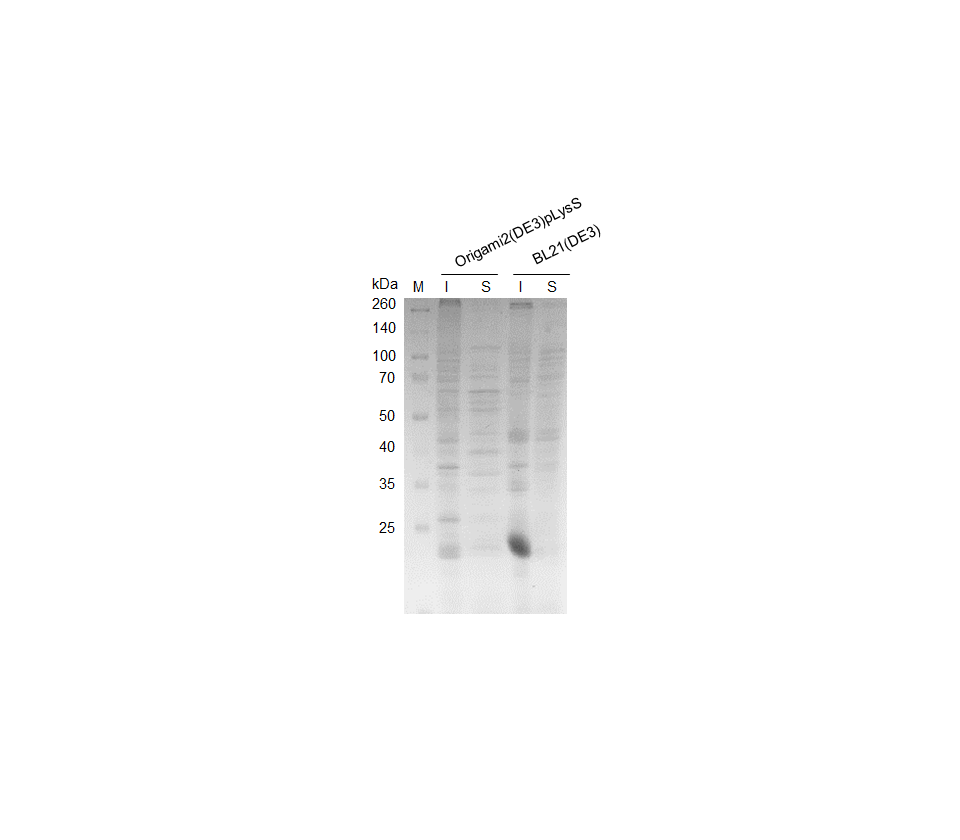

Supplement: S3 Fig — Trx-AfusinC expression was induced with 0.5 mM IPTG for 3 h at 37 °C E. coli Origami2(DE3)pLysS and BL21(DE3). The crude extract was centrifuged, and insoluble (I) and soluble (S) fractions were analysed. Protein samples resolved by 12% Glycine SDS-PAGE. The gel was stained with Coomassie blue R-250. (TIF) [file pone.0205509.s003.tif]
